# Factors leading to effective social participation promotion interventions for people with intellectual disability: a protocol for a systematic review

**DOI:** 10.1186/s13643-021-01716-3

**Published:** 2021-06-04

**Authors:** Andrés Aparicio, Paulina Arango, Rosario Espinoza, Vicente Villate, Marcela Tenorio

**Affiliations:** 1 Millennium Institute for Caregiving Research, República 217, Santiago, Región Metropolitana Chile; 2 Universidad de los Andes, Monseñor Álvaro del Portillo 12455, Las Condes, Región Metropolitana Chile

**Keywords:** Intellectual disability, Social participation, Intervention, Advocacy

## Abstract

**Background:**

People with intellectual disabilities have been historically excluded from decision-making processes. Previous literature indicates that increasing social participation may be an effective way to address this exclusion, but no systematic review of interventions designed to increase social participation of people with intellectual disabilities have been conducted. This study aims to identify and organize the factors associated with interventions that increase the social participation of people with intellectual disabilities and to provide a set of best of practices for future interventions.

**Methods/design:**

The databases Web of Science, Scopus, LILACS, and PubMed will be searched for articles from January 2004 onwards; grey literature search will be identified through searching additional databases (such as Google Scholar and EBSCO databases). Randomized controlled trials, nonrandomized controlled trials, and controlled pre–post studies will be included. Noncontrolled pre–post studies will also be included. Observational or qualitative studies will be excluded. The primary outcomes are measures of social participation. Secondary outcomes include measures of well-being, stigma, knowledge about rights, and advocacy processes. Two reviewers will independently screen articles, extract relevant data, and assess the quality of the studies. We will provide a meta-analysis of included studies if possible, or a quantitative narrative synthesis otherwise.

**Discussion:**

This systematic review will add to our understanding of effective social participation interventions for people with intellectual disability. It will allow us to identify and organize which factors lead to an increase in social participation and help us define a set of best practices to be followed by future interventions.

**Systematic review registration:**

PROSPERO CRD42020189093

**Supplementary Information:**

The online version contains supplementary material available at 10.1186/s13643-021-01716-3.

## Background

People with disabilities (PWD) have been historically excluded from decision-making processes, and they have been pushed to the role of subjects of care instead of full citizens with rights, with freedom for choosing when and how to participate in society. This situation has been slowly improving since the United Nations (UN) published the Convention on the Rights of Persons with Disabilities (CRPD) in 2006. As one of the principles of the Convention is that of “full and effective participation and inclusion in society” [1], signatory countries have been trying to implement rights-based intervention programs aiming to facilitate the participation of PWD with varying degrees of success. We are interested in the particular case of interventions involving people with intellectual disability (ID) as they tend to face stronger barriers against participation than other PWD [2].

ID is a neurodevelopmental condition characterized by differences in cognitive development and adaptive behavior as determined by standardized assessment procedures [3, 4]. While self-advocacy groups have had positive impacts for people with ID [5], they still face barriers to participation due to stigma related to differences in verbal communication and learning difficulties, along with the kinds of support they require [6–8]. Adults with ID want to be heard, to participate in their life choices, to be treated as adults and accepted as individuals [2]. Research suggests that people with ID need tools to empower them and spaces where they can learn about their rights because they are not usually afforded the opportunities to participate [9].

Social participation is a tool of change and social justice that facilitates taking into account the needs of stakeholders in a given situation and increases social inclusion. Previous studies have confirmed that there is a direct relationship between participation, quality of life, and well-being at personal, family, and social levels and that it improves self-esteem, trust, happiness, and mental health and elevates the effective contribution of people with ID to society [10, 11]. Given the relevance of social participation for people with ID, we are interested in finding interventions designed to improve it. This review aims to identify and organize the factors associated with interventions that increase social participation of people with ID and deliver the foundation for a set of best practices in social participation interventions.

As the concept of social participation has been used in different ways in recent years, we will follow previous work to clarify it [12–14] and operationalize social participation as the performance of actions by an individual, interacting with others, that contribute to themselves and others, and occurring at community and sociopolitical levels. This definition will guide our search and provide a framework for categorizing interventions.

The goal of this systematic review is to identify personal, social, and methodological factors that improve the effectiveness of social participation promotion interventions for people with intellectual disability. Our secondary goal is to delineate a set of best practices for social participation promotion interventions based on the evidence collected. To this end, this proposed review will attempt to answer the following questions:

1. Do social participation promotion interventions have an effect on subsequent social participation of people with ID?
  - Which kind of interventions appear to increase social participation?
    i. Do participants in these interventions share any common personal factors?
    ii. Do participants in these interventions share any common social factors?
    iii. Do these interventions share any common methodological factors?
  - Is there a common set of personal, social, and methodological factors shared by effective interventions?

## Methods/design

This protocol has been registered within the PROSPERO database (registration number CRD42020189093). It is being reported in accordance with the guidance provided in the Preferred Reporting Items for Systematic Reviews and Meta-Analyses Protocols (PRISMA-P) statement [15].

### Eligibility criteria

Studies will be selected according to the criteria outlined below.

#### Study designs

We will include randomized controlled trials, nonrandomized controlled trials, and controlled pre–post studies that compare an intervention designed to improve social participation of people with ID. We will also include noncontrolled pre–post studies. We will exclude observational or qualitative studies.

#### Participants

We will include studies about promotion interventions involving adults with ID in three age groups: young adults (18 years old to 25 years old), adults (25 years old to 65 years old), and older adults (65 years old and over). We will include studies about interventions where at least 50% of the participants are people with ID. We will include studies that recruited people regardless of gender, ethnic group, medical diagnosis, or multiple diagnoses, as long as they have been identified as adults with ID.

#### Interventions

We are interested in social participation promotion interventions involving adults with ID, taking a broad perspective. We will consider both offline and online interventions. We will include studies reporting either positive or negative impact on social participation or no impact at all.

We broadly define social participation promotion interventions as those attempting to increase the social participation of people with ID, that is, attempting to increase their performance of actions contributing to themselves and others, in the community and sociopolitical levels. We will exclude studies reporting interventions that do not fit the previous definition.

#### Outcomes

The primary outcomes will include any measures of social participation reported by the studies: employment rate, weekly hours of community work, membership in advocacy networks, involvement in political activities, etc. Secondary outcomes will include measures of well-being, stigma, and knowledge about rights and advocacy processes if they are available. Outcomes will be collected as reported. We will consider creating an aggregate index of effectiveness if the collected studies warrant it.

#### Timing

Studies will be selected for inclusion regardless of the duration of the intervention. Outcomes will be grouped into three categories to represent short-term (less than 6 months), medium-term (between 6 and 12 months), and long-term outcomes (more than 12 months).

#### Setting

There will be no restrictions by the type of setting.

#### Language

We will include articles reported in Spanish, Portuguese, and English.

### Information sources and search strategy

The primary source of literature will be a search of the following electronic databases (from January 2004 onwards): Web of Science Core Collection, PubMed/MEDLINE, Scopus, and LILACS. The secondary source of potentially relevant material will be a search of the grey or difficult to locate literature, including EBSCO databases and Google Scholar. We will perform hand-searching of the reference lists of included studies, relevant reviews, or other relevant documents. We will develop a search strategy based on keywords related to intellectual disability (and historic forms of naming the concept) and social participation. No study design, date, or language limits will be imposed on the search, although only articles in Spanish, Portuguese, and English will be included. A draft search strategy for Web of Science is provided in Additional file 2.

### Selection process

Our review team is made up of three senior researchers, three young researchers, and five junior researchers. Two of the senior researchers will be arbitrators. We will follow a two-stage screening process. First, we will screen using the titles and abstracts yielded by the initial search. Each record will be independently screened by two reviewers who will vote to include or exclude it according to the inclusion criteria. Any disagreements between reviewers will be resolved by the review team during a weekly meeting. If no agreement is reached, the record will be marked as uncertain and retained for the next step. Two senior reviewers will check a report about all titles appearing to meet the inclusion criteria, and those where there is uncertainty. Second, full texts of all retained articles will be procured and assessed independently to decide whether they will be included in the data extraction step. Each record will be processed by two reviewers who will vote to include or exclude it according to the inclusion criteria. Any disagreements will be solved through a discussion between the two reviewers and, if necessary, the involvement of a third reviewer. In case it is needed, we will seek additional information from study authors to determine eligibility. We will record the reasons for excluding studies. No reviewers will be blind to the journal titles, the study authors, or institutions.

### Data extraction and management

The team will design and pilot standardized forms for data extraction. The information to be extracted includes: study methodology, study setting, study population, participant demographics, baseline characteristics, intervention details, outcome measurements and timing, and information for the assessment of risk of bias. Due to the broad perspective for interventions, the forms may require constant refinement during the data extraction process to include previously unidentified outcome measures. Nine reviewers will participate in the data extraction process. To ensure consistency, we will conduct calibration exercises before starting the review. For each study, data will be extracted independently by two reviewers. Reviewer’s disagreement will be resolved by discussion, and one of two arbitrators (MT or PA) will adjudicate unresolved disagreements. We will contact corresponding authors if necessary, to seek out further information.

We will use Mendeley to manage the references. We will use Covidence to carry out the review.

### Risk of bias in individual studies

The methodological quality of each of included study will be assessed independently by two reviewers. As we will be including both randomized and nonrandomized study designs, we will use Cochrane’s respective risk of bias tools: RoB for randomized designs and ROBINS-I for nonrandomized designs [16, 17]. For each study, all risk of bias domain will be rated and categorized as low, high, or uncertain. If there is disagreement between reviewers, it will be solved by discussion and one of two arbitrators (MT or PA) will adjudicate unresolved disagreements.

### Measures of effect

Whenever possible, we will calculate measures of effect. For studies with a separate control group, we will express the intervention effect as either a risk ratio (for dichotomous variables) or standardized mean differences (for continuous variables) with corresponding 95% confidence intervals. For studies without a separate control group and at least three measures before and three measures after the intervention, we will express the effect as standardized level and slope differences of pre–post regression lines [18]. For studies without a separate control group and less than three measures pre–post the intervention, we will use the standardized mean difference between the latest measure before intervention and the earliest measure after intervention.

### Evidence synthesis

We will meta-analyze study results if there are more than three studies presenting original data on the primary outcomes and the I^2^ statistic does not exceed 75% [19]. Study results will be pooled for meta-analysis using a random effects model and presented in forest plots. Publication bias will be explored with funnel plots if enough studies are identified. Subgroup analyses by age group and intervention design will be conducted if sufficient data are available.

If no meta-analysis is possible, we will undertake a quantitative narrative synthesis guided by the Systematic review without meta-analysis guidelines [20]. Studies will be grouped for synthesis by age group and intervention design. Quantitative effect sizes will be presented in tables and will not be combined for presentation. Heterogeneity will be explored using the I^2^ statistics or, if not possible, by sorting studies according to their characteristics. We will prioritize studies depending on the assessed risk of bias, sample and effect sizes, and pertinence to the research questions. For each synthesis group, we will provide a description of synthesized findings accounting for the certainty of results and their assessed risk of bias.

### Confidence in cumulative evidence

We will use the Grading of Recommendations Assessment, Development and Evaluation (GRADE) framework to assess confidence [19]. Evidence quality is classified into high, moderate, low, and very low. This assessment is based on study type, its limitations, inconsistencies in results, and potential biases.

Given our interest in identifying the characteristics of interventions that have a positive impact in social participation, we will emphasize identifying and classifying which interventions characteristics may be factors for this impact. We will weigh the relative strength of each factor by taking into account how many studies provide evidence for it and the qualities of those studies. A summary of these findings will be provided as an attempt to characterize a set of best practices for social participation interventions for people with ID.

## Discussion

We will amend our published PROSPERO review protocol (CRD) if any changes are needed as we conduct the systematic review, and we will report these changes in the final manuscript. We will submit the systematic review for publication in a high-impact peer-reviewed journal. We will present the results at conferences in the areas of intellectual disability, social participation, and advocacy; results will be disseminated to international policymakers through the academic and public policy network of the Millennium Institute of Caregiving Research (MICARE).

We expect the collected quantitative data to vary between studies since we are operationalizing social participation broadly. Due to the nature of the population of interest and the diversity of possible intervention approaches, we acknowledge that the heterogeneity of studies identified may impede conducting a meta-analysis. Despite these potential limitations, we believe the proposed review addresses an important issue. The barriers against the participation of people with ID tend to be higher than for other PWD and, because of this, they are at risk of being excluded from decision-making processes involving their well-being, and from society at large. Increasing social participation of people with ID depends on facilitating their participation in activities at the community and sociopolitical levels aiming to perform actions that benefit themselves and others. Determining which factors lead to interventions that increase social participation is important to guide the design, development, and implementation of better interventions and public policy. By identifying and categorizing these factors, this systematic review will help create a set of best practices for future interventions and public policy and, hopefully, improve the social inclusion of people with ID.

## Supplementary Information

**Additional file 1.** PRISMA+P Checklist.

**Additional file 2.** Literature search strategy for systematic review of factors leading to effective of social participation promotion interventions for people with intellectual disability.

## Data Availability

Not applicable

## Abbreviations

PWD: People with disabilities
UN: United Nations
CRPD: Convention on the Rights of Persons with Disabilities
ID: Intellectual disability
RoB: Cochrane’s Risk of Bias Tool for Randomized Trials
ROBINS-I: Cochrane’s Risk of Bias in Non-Randomized Studies or Interventions
GRADE: Grading of Recommendations Assessment, Development and Evaluation
