# Supplementary material for: Factors leading to effective social participation promotion interventions for people with intellectual disability: a protocol for a systematic review
Source: Syst Rev. 2021 Jun 4;10:165. doi: 10.1186/s13643-021-01716-3 (PMC8176727; doi:10.1186/s13643-021-01716-3)
Supplement: Supplementary file 2 — Additional file 2. Literature search strategy for systematic review of factors leading to effective of social participation promotion interventions for people with intellectual disability. [file 13643_2021_1716_MOESM2_ESM.pdf]

Additional file 2

Literature search strategy for systematic review of factors leading to effective social participation promotion interventions for people with intellectual disability

Database 1 (Web of Science) search strategy

(

TS=(intellectual disabilit* OR learning disabilit* OR mental retardation OR developmental disabilit* OR neurodevelopmental disorder* OR developmental disorder* OR autism OR down syndrome OR trastorno* del neurodesarrollo OR trastorno* del desarrollo OR discapacidad intelectual OR discapacidad de aprendizaje OR retardo mental OR retraso mental OR autismo OR síndrome de down)

AND

TS=(social participation OR political participation OR community participation OR advocacy OR empowerment OR self-determination OR participación social OR autodeterminación OR vocería OR empoderamiento)

AND

TS=(intervention OR workshop OR course OR program* OR intervención OR taller OR curso)

) OR (

TI=(intellectual disabilit* OR learning disabilit* OR mental retardation OR developmental disabilit* OR neurodevelopmental disorder* OR developmental disorder* OR autism OR down syndrome OR trastorno* del neurodesarrollo OR trastorno* del desarrollo OR discapacidad intelectual OR discapacidad de aprendizaje OR retardo mental OR retraso mental OR autismo OR síndrome de down)

AND

TI=(social participation OR political participation OR community participation OR advocacy OR empowerment OR self-determination OR participación social OR autodeterminación OR vocería OR empoderamiento)

AND

TI=(intervention OR workshop OR course OR program* OR intervención OR taller OR curso)

)
